# Towards a Portable Model to Discriminate Activity Clusters from Accelerometer Data

**DOI:** 10.3390/s19204504

**Published:** 2019-10-17

**Authors:** Petra Jones, Evgeny M. Mirkes, Tom Yates, Charlotte L. Edwardson, Mike Catt, Melanie J. Davies, Kamlesh Khunti, Alex V. Rowlands

**Affiliations:** 1 Leicester Diabetes Centre, University Hospitals of Leicester, Leicester LE5 4PW, UK; melanie.davies@uhl-tr.nhs.uk (M.J.D.); kk22@leicester.ac.uk (K.K.); 2 Diabetes Research Centre, University of Leicester, Leicester General Hospital, Gwendolen Road, Leicester LE5 4PW, UK; ty20@leicester.ac.uk (T.Y.); ce95@leicester.ac.uk (C.L.E.); alex.rowlands@leicester.ac.uk (A.V.R.); 3 Department of Mathematics, ATT 912, Attenborough Building, University of Leicester, University Road, Leicester LE5 4PW, UK; em322@leicester.ac.uk; 4 NIHR Leicester Biomedical Research Centre, Leicester General Hospital, Gwendolen Road, Leicester LE5 4PW, UK; 5 Institute of Neuroscience, Henry Wellcome Building, Faculty of Medical Sciences, Newcastle University, Newcastle upon Tyne NE2 4HH, UK; michael.catt@newcastle.ac.uk; 6 Alliance for research in Exercise, Nutrition and Activity (ARENA), Sansom Institute for Health Research, Division of Health Sciences, University of South Australia, Adelaide SA 5001, Australia

**Keywords:** unsupervised, machine learning, physical activity, clustering, wrist-worn, accelerometer, walking

## Abstract

Few methods for classifying physical activity from accelerometer data have been tested using an independent dataset for cross-validation, and even fewer using multiple independent datasets. The aim of this study was to evaluate whether unsupervised machine learning was a viable approach for the development of a reusable clustering model that was generalisable to independent datasets. We used two labelled adult laboratory datasets to generate a k-means clustering model. To assess its generalised application, we applied the stored clustering model to three independent labelled datasets: two laboratory and one free-living. Based on the development labelled data, the ten clusters were collapsed into four activity categories: sedentary, standing/mixed/slow ambulatory, brisk ambulatory, and running. The percentages of each activity type contained in these categories were 89%, 83%, 78%, and 96%, respectively. In the laboratory independent datasets, the consistency of activity types within the clusters dropped, but remained above 70% for the sedentary clusters, and 85% for the running and ambulatory clusters. Acceleration features were similar within each cluster across samples. The clusters created reflected activity types known to be associated with health and were reasonably robust when applied to diverse independent datasets. This suggests that an unsupervised approach is potentially useful for analysing free-living accelerometer data.

## 1. Introduction

Given the beneficial effects of physical activity for health [1], an accurate assessment of physical activity is important. Therefore, small-body worn high-resolution accelerometers are now routinely used in large-scale surveys such as UK Biobank to objectively assess physical activity [2]. Methods used to explore these data are still relatively simple, relying on summary data rather than harnessing the full potential of high resolution (up to 100 Hz) data. This is unfortunate as a more refined approach to accelerometer analytics could facilitate the development of personalised interventions tailored to how types of physical activity are clustered. For example, the automatic detection or classification of broad types of physical activity from accelerometer data could provide valuable information about optimal lifestyle and exercise patterns for the prevention of chronic disease, assist with emerging precision medicine development, and the refinement of programmes for specific health needs (e.g., maintaining cardio-metabolic health) [3,4,5,6]. 

Supervised machine learning models are frequently used to train an automatic classifier to identify activity type from accelerometer data. Studies to date have either focused on the intensity of the activity [7] or activity type with combined categories of running/walking and standing/moving [8]. The models do not always transfer well to new datasets and experience great variability of performance [9], with performance further reduced when models are applied to free-living datasets [10,11]. Therefore, robust cross-validation of models is essential. However, relatively few studies have used more than one dataset to develop and validate these models. Leave-one-out cross-validation [10] is frequently used with only a few studies using an independent dataset for cross-validation [7,8] to confirm generalisability. 

In Kerr et al. [8], a random forest model classified all free-living behaviours with 80% accuracy based on hip accelerometer data. The model was applied to three diverse datasets: these consisted of two female researchers aged under 30, the second of 40 cyclists with a mean age of 36 of which 70% were male, and finally, the third dataset consisted of 36 overweight women with a mean age of 56. Within each dataset, accuracy was evaluated based on leave-one-participant out cross-validation; across datasets, the accuracy was evaluated based on training on an entire dataset and applying that model on each participant from another dataset. Leave-one-out cross-validation and use of an independent dataset for cross-validation were directly compared by Montoye et al. [7] using two wrist accelerometer datasets collected during simulated free-living conditions. There were inconsistencies between the leave-one-subject out cross-validation and independent dataset cross-validation when evaluating the performance of the machine learning models for the prediction of activity intensity. This implies that the leave-one-out method is insufficient for testing how a model will work with a new population [7]. This highlights the importance of including free-living data and using an independent dataset for cross-validation when developing and evaluating machine learning models. Furthermore, the Kerr model also relies on time-smoothing (i.e., using information from neighbouring minutes to improve predictions using a Hidden Markov Model in conjunction with random forests), which requires additional computational time.

Whilst results from the cross validation in independent datasets from the random forest model in Montoye [7] were encouraging (77.3–78.5%) in classifying activities by intensity (sedentary, light, moderate, or vigorous), the study was limited to two datasets consisting of a total of 63 participants with the second dataset showing little variability in age and was based on data collected in a simulated free-living setting within a research laboratory rather than actual free-living data. 

Supervised machine learning models are reliant on labelled data being available (i.e., where it is known what a person is doing), which can be both time-consuming and costly to acquire, particularly for free-living data [10]. An alternative approach is to use unsupervised machine learning models, which infer patterns from an accelerometer dataset without reference to labelled classes. Unsupervised machine learning can provide insights into the underlying structure of data, automatically separate the dataset into clusters of behaviours exhibiting similar movement patterns and facilitate greater understanding of why certain activities are grouped together through cluster analysis [12]. As with supervised learning, ideally, models should be tested on independent datasets to determine whether the cluster structure is generalisable. However, as there is no need for labelled data to train a model, unsupervised machine learning models can be developed on free-living data meaning that they do not require expensive and time-consuming calibration studies [9].

Recently, van Kuppevelt et al. [9] applied an unsupervised approach to classify physical activity intensity from wrist-worn accelerometer data from free-living children. As their aim was to classify activity intensity, not activity type, the input metrics were restricted to five-second averages of resultant acceleration, which was correlated with activity energy expenditure [13], and accelerometer orientation. Their results were reproducible when trained on a sub-sample from the same dataset and showed face validity in terms of the duration and intensity of states identified in their Markov model and relative to the cut-point approach. However, as the authors acknowledged, due to the lack of a gold standard for the intensity of physical activity that is feasible for large free-living studies, criterion validity could not be tested. 

Application of unsupervised approaches to the features from the high-resolution acceleration signal has potential for the classification of activity types. However, in order to understand this approach, it is first necessary to determine whether it works to differentiate known labelled activities and to describe the activity types contained in the clusters generated. 

Our overall aim was to evaluate whether it is possible to use an unsupervised machine learning approach to create a portable (i.e., generalisable and reusable) clusters model that can distinguish between broad categories of physical activity that encompass waking hours and can be easily reapplied across different datasets using stored settings. If successful, this method of approach could be used to develop a general model for the analysis of free-living datasets created with accelerometer devices with similar characteristics and worn at the same body-site. 

Specifically, we aimed to:

1. Fit an unsupervised machine learning model based on two combined labelled accelerometer datasets;
2. Test the fitted model on three independent labelled datasets, one of which is a free-living dataset; and
3. Assess which activity types were clustered together using the ‘ground truth’ information for these datasets

## 2. Materials and Methods

**Figure 1:**
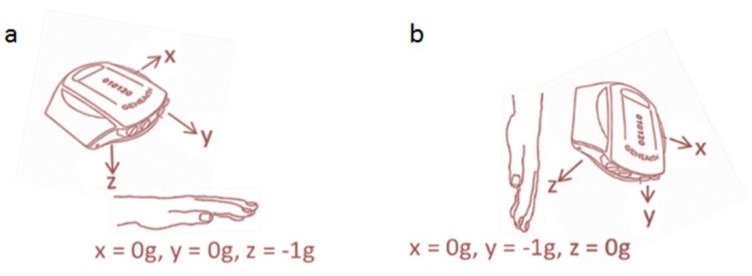
Orientation of the GENEA/GENEActiv axes when worn on the non-dominant wrist with the hand (**a**) level and (**b**) hanging vertically.

Our data were gathered from five studies where accelerometer data were acquired at a sampling frequency of between 80–100 Hz for three axes using the GENEA^TM^ or GENEActiv^TM^ devices [14,15,16,17] (see Table 1 for details of each sample). Participants in all studies gave their written informed consent. Ethics approval was obtained from the Ethics Committees of the School of Sport and Health Sciences, University of Exeter (Development sample 1 and Independent sample 1) and Loughborough University (Development sample 2 and Independent samples 2 and 3). The GENEA is an acceleration sensor developed by Unilever Discover (Colworth, United Kingdom), while GENEActiv is the commercially available version manufactured and distributed by ActivInsights Ltd. (Kimbolton, Cambridgeshire, UK). Both are triaxial MEMS (micro-electro-mechanical system) acceleration sensors housed in a small lightweight casing; the dynamic range of the GENEA is ±6*g* and that of the GENEActiv is ±8*g*. The sampling frequency of the GENEA ranges from 10 to 160 Hz and that of the GENEActiv from 10 to 100 Hz. Our goal was to develop a model that was generalisable to monitors worn on either wrist, therefore data from both wrists were combined for testing. Figure 1 shows the orientation of the axes when worn on the non-dominant wrist with the hand (a) level and (b) hanging vertically. 

The orientation angles of the acceleration axes were relative to the horizontal plane and were calculated as described by van Kuppevelt et al. [9]:

(1) $${angle}_{x}=\left(\tan^{-1}\frac{{acc}_{x}}{\sqrt{{{acc}_{y}}^{2}+{{acc}_{z}}^{2}}}\right)\cdot \frac{180}{\pi }$$

(2) $${angle}_{y}=\left(\tan^{-1}\frac{{acc}_{y}}{\sqrt{{{acc}_{x}}^{2}+{{acc}_{z}}^{2}}}\right)\cdot \frac{180}{\pi }$$

(3) $${angle}_{z}=\left(\tan^{-1}\frac{{acc}_{z}}{\sqrt{{{acc}_{x}}^{2}+{{acc}_{y}}^{2}}}\right)\cdot \frac{180}{\pi }$$

### 2.1. Development Sample 1 (Laboratory Adult 1)

Each participant completed an ordered series of 10–12 semi-structured activities, see Esliger et al. [15] for full details. In brief, the activities included lying, standing still, seated computer work, treadmill walking (4 km/h, 5 km/h, 6 km/h), outdoor brisk walk (6 km/h), walking up and downstairs, two household activities (randomly selected for each participant from window washing, shelf stacking and sweeping), one treadmill run (8 km/h, 10 km/h, or 12 km/h), and an optional outdoor run (10 km/h). The lying activity was performed for 10 min, whereas all other activities were performed for around 4.5 min. 

### 2.2. Development Sample 2 (Laboratory Adult 2)

Each participant completed a series of activities, as detailed previously [14]. Activities included lying (in various positions including flat on back, flat with legs bent, or to the side with legs either bent or straight), sitting (with different foot or leg positions) with and without upper body movement (computer, and mobile phone games), household activities (washing up, cleaning/dusting, or sweeping), and finally, a self-paced corridor walk. All activities/postures were five minutes in duration.

### 2.3. Independent Sample 1 (Laboratory Child)

Children carried out five activities including lying, seated DVD watching, treadmill walking (4 km/h and 6 km/h) and treadmill running. Lying was performed for ten minutes, with three minutes for the other activities. See Phillips et al. [16] for details.

### 2.4. Independent Sample 2 (Laboratory Adult 3)

The activities of the participants included sitting (engaged in various activities including eating, talking, reading the newspaper, and computer activities whilst seated), standing still, light walking, and household (consisting of dusting, sweeping, and washing up) each of five to ten minutes’ duration. See Rowlands et al. [17] for the full details.

### 2.5. Independent Sample 3 (Free-Living Adult)

These data were taken from a larger dataset investigating the equivalency of physical activity outputs from a range of accelerometers during free-living [12]. For the current analysis, data were taken from the GENEActiv worn on the non-dominant wrist and the activPAL (PAL Technologies Ltd., Glasgow, UK) worn concurrently on the thigh for two days of free-living. The activPAL activity types of sedentary, standing, and stepping were used as a criterion to label the GENEActiv data. The data were analysed in two ways: (a) using labelled data to identify the proportion of each activity type within each cluster of the model as per the laboratory samples, but for the activPAL activity types of sedentary, standing, and stepping only (*N* = 6); and (b) comparing the total daily time identified in the clusters by the model to the activPAL criterion (*N* = 8). For (a), three hours of matched data were selected from the free-living data (between 08:00–11:00 for three participants and between 15:00–18:00 for three others), making a total of 18 h. Three hours was selected to include a range of activities (e.g., self-care, eating, transport, ambulation, work), but also keep files at a manageable size for the labour-intensive job of manually detecting all transitions from the activPAL event file to label each corresponding GENEActiv file. For (b), twenty-four hours of matched data were selected from the free-living data (between 00:00–24:00 for eight participants), making a total of 192 h. This was to give context to the results as it reflects the way the model would likely be applied with free-living data.

### 2.6. Activity Labels 

Across the laboratory datasets, ground truth information was available for nine types of physical activity (lying, seated, standing, household, indoor walking, outdoor brisk walking, treadmill walking, stairs, running). For the free-living dataset, ground truth information for sedentary, standing, and stepping was estimated from the activPAL as detailed above. As described, we applied our model to both a labelled sample of 3 h data for six participants and 24 h data for eight participants.

### 2.7. Pre-Processing of the Accelerometer Signal 

To fit the unsupervised machine learning model to the data and evaluate the purity of the clusters relative to the ground truth, we began by annotating our four laboratory time series data with activity labels and discarded thirty seconds of data from the beginning and before the end of each labelled activity to eliminate the transitional data. For the free-living data, the activPAL event file was used to identify transitions between sedentary, standing, and stepping. We utilised those time ranges within Python (Python Software Foundation, https://www.python.org/), [18] using a purpose-built script, to select a 24 h time-range and generate frequency and time domain features. These initial features were based on popular choices from other studies (see Supplementary Tables S1 and S2). Acceleration features were extracted from all five datasets using ten second sliding windows, with no overlap. Two frequency domain features were extracted. These were the dominant frequency and power of dominant frequency based on a single-dimensional signal magnitude vector ($\mathrm{SMV}=\sqrt{X^{2}+Y^{2}+Z^{2}}$) also referred to as the resultant, or ENMO (Euclidean Norm Minus One) which is ENMO=max{0, SMV−1) [11]. Negative ENMO values were flattened to zero. Further time domain features were extracted including the max, min, mean, median, 10th percentile, 75th percentile, 90th percentile, standard deviation, and variance for each axis. In addition, mean, max, and median for ENMO and the min, max, mean, and median for the orientation angles for each of the three acceleration sensors relative to the horizontal plane were extracted. When inactive (i.e., the only acceleration measured is due to gravity), these angles are an estimate of wrist orientation. We used the absolute value of the correlation coefficient between the feature and the class vector (i.e., type of physical activity) to select the most relevant features and considered coefficients less than 0.12 as weak. This approach selected 24 features (r = 0.12–0.53), presented in Table 2.

Our purpose was an exploratory analysis and we aimed to ascertain which features met the threshold for clustering in the development dataset, which, by definition, was comprised of activity types across the intensity range and representative of daily living [14,15]. Pre-processing also included the MinMax normalisation. 

### 2.8. Unsupervised Machine Learning Using k-Means 

K-means [19] has been a popular clustering method for more than sixty years, remaining one of the key machine learning models that is included in all major statistical packages (e.g., WEKA, R, SPSS, STATA). In each instance, the number of clusters (K) is either known, presumed or indicated beforehand (a number of techniques exist including the Elbow Method [20], Silhouette Score [21], and Calinski-Harabasz [22] to assess an optimal number for K) [23]. Here, we used the Nguyen et al. [24] approach of over-clustering where the number of clusters (ten) was greater than the number of actual or expected classes (nine, i.e., the number of activity types in the development dataset). The centroids of each cluster were initialised randomly, or as here with K++ [25]. 

K-means alternates two steps until convergence. The first step is the association of each data point x with the closest centroid $\mu_{j}$. Let us denote the set of points in cluster i as $C_{i}$:

(4) $$C_{i}=\left\{x:\|x-\mu_{i}\|\leq \|x-\mu_{j}\|\right\}.$$

The second step recalculates the centroids of each cluster to minimise the sum of squared Euclidean distances from the data points of this cluster to the cluster centroid

(5) $$\mu_{j}=\frac{1}{\left|C_{j}\right|}\sum_{x\in C_{j}}x$$

where $\left|C_{i}\right|$ is the number of points in the cluster. This two-step algorithm minimises the sum of squared Euclidean distances from each data point to the nearest centroid. 

The two development datasets were used to fit the model. Ground truth information was available for nine types of physical activity. The trained clustering model (including K, the hyper-parameter settings, and the centroids for each cluster) was stored using the Python pickle module and reapplied blind on the two independent laboratory datasets and the free-living accelerometer dataset. Use of the same model is essential. If the same k-means algorithm is applied to successive datasets, it will calculate different clusters for each database. Such clusters can better describe each individual database but are useless for generalisation purposes. All machine learning algorithms were carried out using the sklearn library [18] in Python.

### 2.9. Evaluation of the Model 

The clustering results from the development datasets were examined to determine how the nine activities were spread across the ten clusters. Based on the clustering observed, to evaluate the models, we collapsed the ten clusters into broad activity type categories with similar properties. These activity type categories were subsequently used to evaluate the clusters relative to the ground truth activity labels and by measuring the combined average cluster purity and average event purity (*ACEP*). The performance of the clustering model was evaluated using a purity matrix to show the proportion of the total instances of each physical activity class within each cluster. This approach has been used in other accelerometer clustering papers [26]. Performance was assessed for the two development datasets combined with each of the independent datasets. We also evaluated cluster purity (*ACP*), a measure of the extent to which the categories contain a single dominant class [27]; average event purity (*AEP*), the proportion of a class found within a cluster relative to that found in other clusters; and combined average cluster purity (*ACEP*). Let us denote $n_{ij}$ as the number of events j in cluster i (number of points of class j in cluster i), $N_{e}$ is the total number of different events (classes), $N_{c}$ is the number of clusters, $nc_{i}=\sum_{j=1}^{N_{e}}n_{ij}$ is the number of elements in cluster i, $ne_{j}=\sum_{i=1}^{N_{c}}n_{ij}$ is the number of instances of event (class) j, and $N=\sum_{j=1}^{N_{e}}ne_{j}=\sum_{i=1}^{N_{c}}nc_{i}$ is the total number of instances (points in database).

(6) $$ACP=\frac{1}{N}\sum_{i=1}^{N_{c}}\sum_{j=1}^{N_{e}}\;\frac{n_{ij}^{2}}{nc_{i}},$$

(7) $$AEP=\frac{1}{N}\sum_{j=1}^{N_{e}}\sum_{i=1}^{N_{c}}\;\frac{n_{ij}^{2}}{ne_{j}},$$

(8) $$ACEP=\sqrt{ACP\times AEP}$$

It should be noted that purity has some limitations when working with imbalanced data where the relative sizes of the classes are different, whereas here, there were multiple classes (e.g., lying down and seated work) both included in a category (e.g., sedentary). However, *ACP* and *AEP* still provide a useful rough guide to the extent to which a given category of PA constitutes the bulk of the cluster. Purity was not calculated for the free-living dataset as the standing and stepping ground truth categories could not be aligned with mutually exclusive clusters (i.e., the clusters did not distinguish between standing and slow stepping) and the ground truth did not distinguish between slow and vigorous stepping.

## 3. Results

### 3.1. Determination of Activity Types That Cluster Together and Evaluation of the Model within the Development Dataset

The purity matrix for the development datasets and percentage of time allocated to each cluster by the algorithm is shown in Table 3, illustrating the proportion of the total instances of each physical activity class within each cluster. The ten clusters in Table 3 are labelled A to J and the proportion of each labelled activity that fell in each cluster is indicated. In the development dataset, nearly all running activity (95.7%) fell in cluster J (running activity marked in bold), with sedentary activities (lying and sitting) in clusters A–E (96.3% of lying and 82.0% of sitting), indoor walking in G–H (86%) while treadmill-based (77.8%), brisker outdoor walking (84.5%), and stairs (75.4%) fell primarily into cluster I.

Household activities and standing were difficult to isolate and tended to ‘bleed’ across the set of clusters, although predominantly (71.2% household and 97.6% of standing) fell in the mixed (F) cluster and indoor walking clusters (G–H). Therefore, there were four key identified physical behaviour categories: sedentary (cluster A–E); standing/mixed/slow ambulatory (F–H); brisk ambulatory (I); and running (J).

### 3.2. Evaluation of the Model in Two Independent Laboratory Datasets 

A large proportion (87.3%) of running activity was captured within the running category when applying the model to the first independent dataset (child laboratory: Table 4 (top)), despite the data coming from children rather than adults. A total of 72.0% of lying down and 85.5% of seated were captured within the sedentary category (clusters A–E), although only 70.5% of the children’s treadmill-based walking fell within the ambulatory category (clusters G–I), with only 57.7% in the brisk walking cluster (I).

When applying the development model to the second independent dataset (adult laboratory: Table 4 (middle)) that did not include lying or running, 71.5% of seated was captured within the sedentary category (clusters A–E), 67.9% of standing in the standing/slow ambulatory category (clusters F–H), 83.7% of household in the standing/slow ambulatory category (clusters F–H), and 87.9% of the indoor walking within the slow ambulatory category (clusters G–H).

### 3.3. Evaluation of the Model in a Free-Living Dataset 

The third independent dataset included free-living adult data, which ass analysed in two ways: (a) to determine the proportion of each activity type within each cluster (Table 4 (bottom)), with the ground truth restricted to the activPAL categories of sedentary, standing and stepping; and (b) to compare the total daily time spent in each cluster to the activPAL determined total daily time spent sedentary, standing, stepping, and brisk walking (Table 5 and Table 6).

(a) Proportion of each activity type within each cluster (Table 4 (bottom)): The model separated 58.5% of sitting/lying into the sedentary category (clusters A–E), and 78.2% of stepping into the ambulatory category or the running category (clusters G–J). Adding the mixed cluster (F) to the sedentary category increased the proportion of sedentary activities captured to 70.9%, with no change to the proportion of stepping captured (78.2%).

(b) Comparison of the daily total time (Table 5): The proportion of time during free-living spent in each cluster is shown in Table 5 and in the broader activity type categories in Table 6. Relative to the activPAL, the clustering model underestimated the time spent sedentary (cluster A:E) and overestimated the time spent at a high stepping frequency (brisk walking clusters I:J), Table 6.

### 3.4. Average Cluster Purity across Multiple Datasets

In the laboratory datasets, ACEP (a measure of combined cluster and event purity) was the highest in the running category (cluster J, 0.80), followed by the sedentary category (clusters A–E, 0.65), and weakest in the slow (clusters G–H) and brisk ambulatory (cluster I) categories (0.49–0.50) (see Supplementary Table S3, average cluster purity and event purity). 

### 3.5. Feature Characteristics of the Clusters by Sample

**Figure 2:**
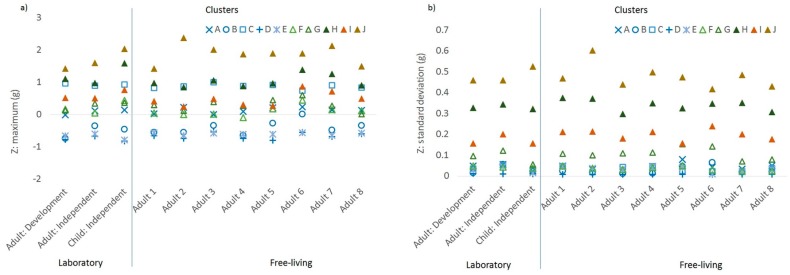
Characteristics of the acceleration features with the highest loadings by cluster and by sample, time domain: (**a**) maximum acceleration in the *Z*-axis and (**b**) standard deviation of acceleration in the *Z*-axis.

**Figure 3:**
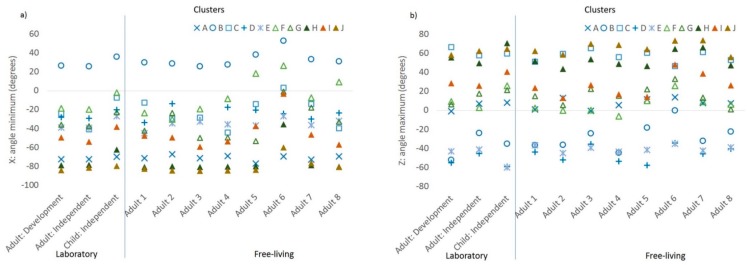
Characteristics of the acceleration features with the highest loadings by cluster and by sample, accelerometer orientation features: (**a**) minimum angle of the *X*-axis acceleration relative to the horizontal plane and (**b**) maximum angle of the *Z*-axis acceleration relative to the horizontal plane.

**Figure 4:**
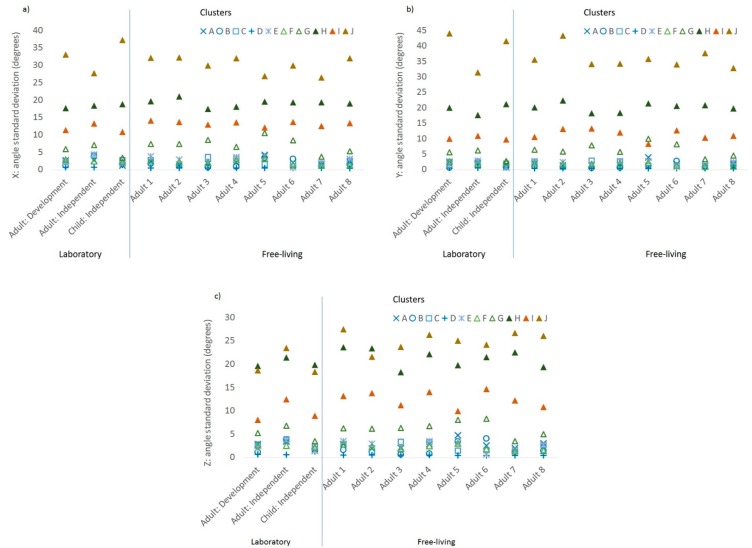
Characteristics of the features with the highest loadings by cluster and by sample, standard deviation of the accelerometer orientation metrics: (**a**) *X*-axis acceleration relative to the horizontal plane, (**b**) *Y*-axis acceleration relative to the horizontal plane, and (**c**) *Z*-axis acceleration relative to the horizontal plane.

The correlation with class for each of the 24 accelerometer features included in the model is shown in Table 2. The mean values of the features with the highest loadings (r > 0.3, *N* = 7) are shown in Figure 2a–g by sample and by cluster. The features with the highest loadings were two acceleration magnitude features: maximum value for Z (Figure 2a), and standard deviation of Z (Figure 2b); and five acceleration orientation features: minimum X angle (Figure 3a), maximum Z angle (Figure 3b), and standard deviation of X, Y, and Z (Figure 4a–c, respectively). In each figure, the samples are on the *x*-axis with the values for the feature characteristics marked for each cluster. Clusters in the sedentary category are denoted in blue, the standing/slow ambulatory category in green, brisk walking category in tan, and running category in brown. The more active clusters are all denoted with triangles. The feature characteristics of the seven features that loaded most highly in the development dataset were fairly consistent for each cluster across samples, as shown by similar values for each cluster category (denoted by colour) and cluster (denoted by symbol).

The five sedentary clusters (denoted in blue) were discriminated most clearly by the maximum acceleration in the *Z*-axis (i.e., whether palm is facing up or down, Figure 2a), minimum X angle (i.e., elevation of the wrist when the palm is vertical, e.g., peak of arm swing when running/walking), Figure 3a), maximum Z angle (i.e., elevation of the wrist when palm is facing up/down, Figure 3b). The more active clusters (triangles: F, G, H, I, J) were most clearly discriminated from sedentary by the standard deviation of the acceleration in the *Z*-axis (palm up/down, Figure 2b) and the orientation of the accelerometer axis relative to the horizontal plane: X, Y, and Z angles (Figure 4a–c).

## 4. Discussion

The aim of the unsupervised approach is not to identify pre-determined categories (e.g., of activity types or intensity), but to identify patterns in the data that clustered together [9]. To facilitate interpretation, we identified which types of activities our model tended to discriminate between. A particular strength of k-means as an unsupervised machine learning model is its portability (i.e., the centroid of each cluster can be stored and reapplied to multiple accelerometer datasets). Theoretically, this enables analogous clusters to be fitted from multiple datasets facilitating comparisons between studies and/or populations.

Application of the unsupervised machine learning model to the relatively simple labelled wrist accelerometer development dataset facilitated discrimination between clusters that reflected activity types. When the stored model was applied to diverse laboratory independent datasets that were also relatively simple, the consistency of activity types within clusters dropped, but remained above 70% for sedentary clusters and above 85% for both the running (children) and ambulatory clusters. Perhaps most notably, while standing mainly fell into the mixed cluster in the development datasets, it tended to bleed into the slow ambulatory clusters in independent dataset 2. Household activities also tended to bleed across clusters. When applied to a small free-living dataset labelled only as sedentary/standing/stepping, the consistency of activity types was similar for sedentary clusters and all ambulatory clusters, although the standing activity again bled across clusters. Total daily time spent in sedentary clusters was lower and time spent brisk walking/running was higher relative to the sedentary and stepping time from the thigh-worn ActivPAL, both with relatively wide limits of agreement. The purpose of these comparisons is to give some context as to the content of the clusters identified by the model; the discrepancies are not surprising given the differences in monitor wear-site and analytical approach and is consistent with other comparisons of methods (e.g., [28]). Notably, the characteristics of the features of the acceleration signal used in the model were similar within each cluster across laboratory-based samples and free-living participants, across diverse populations (e.g., children and adults), and irrespective of whether activity typical of a cluster (e.g., running in the independent adult laboratory sample), were missing. This suggests that application of a stored model can identify clusters that represent similar activities and/or movement patterns, at least in terms of the features used within the model, in children and adults and in laboratory and free-living data. It is important to determine if these clusters are associated with, or predictive of, health outcomes. 

The acceleration features that were selected were largely related to accelerometer orientation and the standard deviation of accelerations. This may have helped the suitability of the model across diverse datasets as these features are likely less impacted by body size than the resultant acceleration, which can differ for a given activity by size (e.g., between children and adults) [29]). The heaviest loading was on features related to the *Z*-axis and on the standard deviation of the accelerometer orientation, likely reflecting the inclusion of data from both wrists in the development of our wrist agnostic model. Inclusion of data from both wrists will have confounded magnitudes of acceleration and, to a lesser extent, the orientation of the vertical axis of acceleration (Y) and the anterior–posterior acceleration (X, e.g., arm swing), but not the *Z*-axis (going into the wrist). This is a limitation in the features available for model development, but it also extends the generalisability of the model and thus external validity. The model should be robust to differences in wear including not only on either wrist, but potentially irrespective of the positioning on the wrist (e.g., wear with the face of the monitor on the inside or outside of the arm).

The model was created somewhat artificially using a laboratory dataset. This is a limitation as behaviours performed in a laboratory setting differ from those performed in a free-living setting [13]). This was deliberate as a ‘first step’ to establish whether an unsupervised approach provides meaningful clusters when applied to accelerometer data. As the clusters produced in this controlled scenario appear meaningful, this provides a strong foundation for applying the approach developed to free-living data. By using laboratory datasets, we were able to (a) start with a simpler clustering task, and (b) assess the robustness of the model on multiple datasets that differed in the population considered, protocol, activities undertaken, laboratory where the study was undertaken, and version of the accelerometer used. Very few studies have undertaken cross-validation using multiple datasets [7,8,16]. That the types of activities contained in the sedentary clusters and the ambulatory clusters in the laboratory datasets broadly agreed with the stepping and standing assessed by the activPAL in the pilot free-living dataset is encouraging. This suggests that meaningful clusters that are comparable between datasets could be obtained from applying this type of approach to free-living accelerometer data.

The magnitude of acceleration alone is a useful metric for classifying moderate and vigorous activity, either through the cut-point [29] or other more data-driven approaches [30]. However, it is not useful for differentiating between types of sedentary and lighter activities, which comprise the majority of the day [28]. The identification of multiple sedentary clusters, in both this study and in van Kuppevelt’s [9] earlier study, that differ predominantly in the acceleration orientation features suggest that the primary advantages of this approach may be the classification of types of sedentary and light behaviour. The results of this study build on our previous research showing how, when a person is inactive, the acceleration orientation metrics from wrist-worn accelerometers can be exploited to determine wrist position, estimate posture, and visualise different types of sedentary behaviours [28]. For example, cluster C was characterised by the palm tending to face up (as shown by positive maximum Z acceleration), cluster A with the palm neither up or down (0 maximum Z acceleration), and the remaining clusters with the palm facing more downwards (negative maximum Z acceleration). The elevation of the wrist when the palm was vertical was consistently positive in cluster B, while the elevation of the wrist when the palm was facing down was consistently positive in cluster C, negative in clusters D–E, and zero in cluster A. Obtaining ground truth information from the detailed type of sedentary behaviour would allow this to be further explored. Meanwhile, the standard deviations of the accelerometer orientation metrics were important features for discriminating between activity and inactivity, particularly between ambulatory activities and sedentary behaviour. These features reflect variability in the elevation of the wrist and are agonistic to the wrist of wear.

This is an exploratory study and there are weaknesses and areas for improvement. For example, it is desirable to minimise the bleeding of non-ambulatory activities such as standing and household activities into the ambulatory clusters. K-means has a bias towards forming spherically shaped clusters and necessitates a choice of K amongst other drawbacks [31]. Given comparatively poor internal measure cluster validation scores (Silhouette Score 0.22–25), which indicate a lower level of cohesiveness and separation between clusters, there is potential for further work on feature selection and engineering to maximise the separation between accelerometer physical activity clusters, perhaps experimenting with additional frequency domain features (e.g., zero-crossings, second dominant frequency, or the ratio between dominant frequencies for current and previous windows) to try to increase cluster separation and cohesiveness. Furthermore, our features were generated on non-overlapping windows; another approach could be to incorporate the stage before and the stage after (i.e., overlapping windows) into feature engineering to see if this facilitated better discrimination of activity types that cluster together. Finally, while the results herein suggest portability, all datasets used the GENEA or the GENEActiv with a sampling frequency of 80–100 Hz. Thus, we have only considered portability between datasets using the same device and not between devices. It is possible that the model may not be portable to datasets using the same device with a lower sampling frequency and/or using a different device. There is evidence for equivalence in outputs between research-grade accelerometer brands [12,17], particularly for frequency domain and orientation features [32,33,34], which suggest that the model may be portable between research-grade devices, but this needs to be confirmed in future research.

## 5. Conclusions 

The clusters created herein reflected activity types that are known to be associated with health (i.e., sedentary and ambulatory activities) [35], and were reasonably robust when the stored model was applied to diverse datasets. This suggests an unsupervised approach is potentially useful for analysing free-living accelerometer data. The model produced was wrist agnostic, simplifying application to large datasets where participants may change the wrist of wear or position of the monitor on the wrist. A key advantage of unsupervised machine learning is the removal of the need for labelled data, which is costly and time consuming to obtain. This work suggests it is possible to use an unsupervised machine learning approach to create a portable (i.e., generalizable and reusable) clusters model that can distinguish between broad categories of physical activity across datasets created with accelerometer devices with similar characteristics and worn at the same body-site. This provides the foundation for further work deploying a k-means unsupervised approach to develop a model on free-living data that can subsequently be stored and re-applied on other datasets, creating comparable clusters between studies and/or populations using a similar accelerometer device. Information on the feature characteristics of the accelerometer signal in activity categories, as presented herein, could aid in the identification of the physical behaviours most likely associated with clusters generated on unlabelled free-living data. Moving forward, a key question will be whether the clusters are associated with health and whether the use of clustering adds value to existing methods of analysing accelerometer data when considering health, free-living behaviours, and behaviour change.

## Figures and Tables

**Table 1:** Characteristics of development and independent datasets.

Dataset	Sample	Participants (N, % Female)	Sampling Rate (Hz)	Monitor	Age (y)	Height (cm)	Mass (kg)	Handedness	Monitor Location
Dev 1	Lab: Adult	60 (62%)	80	GENEA	40–65	176.2 (6.2)	80.6 (11.6)	55R, 5L	Both wrists
Dev 2	Lab: Adult	30 (73%)	100	GENEActiv	20–40	169.4 (0.1)	69.2 (15.3)	27R, 3L	Non-dom.
Ind 1	Lab: Child	41 (59%)	80	GENEA	9–14	150.2 (13.3)	43.0 (11.2)	37R, 2L, 2A	Both wrists
Ind 2	Lab: Adult	23 (70%)	100	GENEActiv	19–42	172.7 (7.9)	73.7 (13.0)	18R, 5L	Non-dom.
Ind 3	Free-Living: Adult (3 h)	6 (33%)	100	GENEActiv	20–29	171.5 (10.9)	73.0 (17.1)	6R	Non-dom.
Ind 3	Free-Living: Adult (24 h)	8 (62.5%)	100	GENEActiv	20–29	166.8 (98.1)	65.4 (11.3)	8R	Non-dom.

Handedness L = left, R = right, A = ambidextrous. Non-dom. = non-dominant wrist. Lab = laboratory dataset. Dev = development dataset, Ind = independent dataset.

**Table 2:** Loading (Pearson’s correlation) on acceleration features included in the model (development dataset).

Acceleration Feature		Loading	Acceleration Feature		Loading
Frequency	Dominant Frequency	−0.271	Angle	X—Minimum	−0.389
Magnitude	X—Minimum	−0.285		X—Median	−0.176
	X—Maximum	0.233		X—Mean	−0.175
	X—Standard Deviation	0.195		X—Maximum	0.220
	Y—Minimum	−0.286		X—Standard Deviation	0.394
	Y—Standard Deviation	0.182		Y—Minimum	−0.241
	Z—Minimum	−0.194		Y—Maximum	0.169
	Z—75th Percentile	0.209		Y—Standard Deviation	0.329
	Z—Maximum	0.358		Z—Minimum	−0.183
	Z—Standard Deviation	0.440		Z—Median	0.124
	Z—Variance	0.262		Z—Mean	0.125
				Z—Maximum	0.340
				Z—Standard Deviation	0.526

**Table 3:** Purity matrix (percentage of each class found within each cluster (A–J)) for the development dataset (two combined adult datasets). (Key statistics highlighted in bold)

						Ambulatory				
Sedentary					Mixed	Slow		Brisk	Running	
A	B	C	D	E	F	G	H	I	J	Class
9.1	3.9	6.9	4.4	12.2	15.7	11.6	7.2	24.2	4.8	% of total time
**12.2**	**24.6**	**22.2**	**16.0**	**21.2**	1.4	2.0	0.2	0.1	0.0	Lying
**23.3**	**0.2**	**13.5**	**8.1**	**37.0**	12.2	2.6	2.4	0.7	0.1	Seated
0.4	0.0	0.6	0.3	0.0	**65.9**	**29.3**	2.4	1.1	0.0	Standing
8.1	0.1	0.8	0.0	1.4	**16.9**	**9.8**	**44.6**	15.6	2.8	Household
2.6	0.1	0.3	0.0	0.6	0.0	**55.4**	**30.6**	4.2	6.3	Indoor walking
0.0	0.0	0.6	0.0	0.1	16.0	1.8	3.5	**77.8**	0.3	Treadmill walking
0.0	0.0	0.0	0.0	0.0	1.4	2.5	9.5	**84.5**	2.2	Brisk outdoor walk
1.4	0.0	0.1	0.0	0.1	17.1	1.8	4.0	**75.4**	0.2	Stairs
0.0	0.0	0.0	0.0	0.0	1.8	0.0	1.2	1.2	**95.7**	Running

**Table 4:** Purity matrices for independent datasets 1 to 3 (percentage of each class found within each cluster (A–J).

						Ambulatory				
Sedentary					Mixed	Slow		Brisk	Running	
Independent Sample 1: Child Laboratory										
A	B	C	D	E	F	G	H	I	J	Class
6.5	13.1	19.9	0.2	5.5	12.3	5.7	2.5	14.7	19.7	% of total time
6.7	24.0	29.3	0.5	11.4	15.6	5.0	3.5	3.1	0.9	Lying
31.1	15.1	38.9	0.0	0.5	8.7	0.3	3.7	1.5	0.3	Seated
0.0	0.0	4.6	0.0	0.0	18.2	0.2	12.6	57.7	6.7	Treadmill walking
0.0	0.0	3.9	0.0	0.0	0.3	0.1	4.5	3.9	87.3	Running
**Independent sample 2: Adult laboratory**										
20.1	7.6	7.5	3.4	1 7.0	3.8	24.3	10.1	4.7	1.7	% of total time
25.5	10.2	8.7	4.6	22.5	4.8	1.9	16.0	5.2	0.5	Seated
14.3	0.0	13.0	0.2	2.4	2.2	53.4	12.3	1.3	0.9	Standing
4.2	0.2	2.1	0.1	1.2	0.4	16.9	66.4	2.1	6.4	Household
0.0	0.0	0.0	0.0	0.2	0.2	62.1	25.7	7.8	4.0	Indoor walking
**Independent sample 3A: Adult free-living (*N* = 6)**										
20.2	2.2	6.2	2.4	15.9	8.6	27.2	6.8	7.4	3.1	% of total time
19.7	3.6	7.5	3.9	23.7	12.4	14.8	2.1	11.4	0.8	Sedentary
26.2	0.2	5.5	0.3	7.7	4.0	38.1	10.4	1.9	5.8	Standing
14.4	0.1	3.3	0.1	1.6	2.3	52.6	17.3	1.5	6.8	Stepping

**Table 5:** Independent dataset 3 (free-living). Percentage of total daily time found in each cluster (A–J).

							Ambulatory				
	Sedentary					Mixed	Slow		Brisk	Running	
	Independent Sample 3b: Free-Living (*N* = 8)										
	A	B	C	D	E	F	G	H	I	J	Class
Mean	19.2	13.1	13.9	6.1	9.7	9.2	16.8	3.5	5.8	2.7	% of total time
SD	11.5	7.7	9.7	2.5	4.7	5.6	4.0	1.6	1.8	1.4	

**Table 6:** Comparison of total daily minutes spent in activity type categories with the activPAL data.

	Clusters A-F	ActivPAL Sedentary	Clusters G-H	ActivPAL Stand/Step	Clusters I-J	ActivPAL High Step
Mean (min)	1024.90 *	1117.54	293.19	280.98	121.17 *	41.49
SD	67.38	64.86	63.59	65.22	38.23	32.19
Bias	−92.6		12.2		79.7	
95% LoA	98.1		132.1		80.2	

* sig different from activPAL (*p* < 0.05).

## Supplementary Materials

The following are available online at https://www.mdpi.com/1424-8220/19/20/4504/s1, Table S1: Summary of Time Domain Features Utilised in Previous Studies, Table S2: Summary of Frequency Domain Features Utilised in Previous Studies, Table S3: Average cluster purity and event purity.
