# Supplementary material for: Towards a Portable Model to Discriminate Activity Clusters from Accelerometer Data
Source: Sensors (Basel). 2019 Oct 17;19(20):4504. doi: 10.3390/s19204504 (PMC6832944; doi:10.3390/s19204504)
Supplement: Supplementary file 1 [file sensors-19-04504-s001.pdf]

sensors

MDPI

# Supplementary Materials: Towards A Portable Model to Discriminate Activity Clusters from Accelerometer Data

**Petra Jones [1,2,*], Evgeny M. Mirkes [3], Tom Yates [2,4], Charlotte L. Edwardson [2,4], Mike Catt [5], Melanie J. Davies [1,2,4], Kamlesh Khunti [1,4] and Alex V. Rowlands [2,4,6]**

[1] Leicester Diabetes Centre, University Hospitals of Leicester, Leicester, LE5 4PW, UK; melanie.davies@uhl-tr.nhs.uk (M.J.D.); kk22@leicester.ac.uk (K.K.)
[2] Diabetes Research Centre, University of Leicester, Leicester General Hospital, Gwendolen Road, Leicester, LE5 4PW, UK; ty20@leicester.ac.uk (T.Y.); ce95@leicester.ac.uk (C.L.E.); alex.rowlands@leicester.ac.uk (A.V.R.)
[3] Department of Mathematics, ATT 912, Attenborough Building, University of Leicester, University Road, Leicester, LE5 4PW, UK; em322@leicester.ac.uk
[4] NIHR Leicester Biomedical Research Centre, Leicester General Hospital, Gwendolen Road, Leicester, LE5 4PW, UK
[5] Institute of Neuroscience, Henry Wellcome Building, Faculty of Medical Sciences, Newcastle University, Newcastle upon Tyne, NE2 4HH, UK; michael.catt@newcastle.ac.uk
[6] Alliance for research in Exercise, Nutrition and Activity (ARENA), Sansom Institute for Health Research, Division of Health Sciences, University of South Australia, Adelaide SA 5001, Australia
* Correspondence: pj100@leicester.ac.uk; Tel.: +44-116-258-4974 (UK)

Received: 4 September 2019; Accepted: 15 October 2019; Published: 17 October 2019

**Table S1.** of Time Domain Features Utilised in Previous Studies.

| Time Domain Features | LDC | Kerr | Kuppervelt | Montoye | Nguyen | Ray | Zhang |
|---|---|---|---|---|---|---|---|
| X, Y and Z Angles Mean | | | | | | | |
| X, Y and Z Angle Max | | | | | | | |
| X, Y and Z Angle Min | | | | | | | |
| X, Y and Z Angle Median | | | | | | | |
| X, Y and Z Axis Correlation | | | | | | | |
| X, Y and Z Axis Mean | | | | | | | |
| X, Y and Z Axis Minimum | | | | | | | |
| X, Y and Z Axis Maximum | | | | | | | |
| X, Y and Z Axis Std. Deviation | | | | | | | |
| X, Y and Z Axis 10th Percentile | | | | | | | |
| X, Y and Z Axis 25th Percentile | | | | | | | |
| X, Y and Z Axis 50th Percentile | | | | | | | |
| X, Y and Z Axis 75th Percentile | | | | | | | |
| X, Y and Z Axis 90th Percentile | | | | | | | |
| X, Y and Z Axis Variance | | | | | | | |
| DWT SMV | | | | | | | |
| DWT SMV1 | | | | | | | |
| ENMO Raw Data | | | | | | | |
| ENMO Mean | | | | | | | |
| ENMO Minimum | | | | | | | |
| ENMO Maximum | | | | | | | |
| ENMO Median | | | | | | | |
| ENMO 25th Percentile | | | | | | | |
| ENMO 75th Percentile | | | | | | | |
| ENMO Std Deviation | | | | | | | |
| ENMO/VM | | | | | | | |
| ENMO/VM Std Deviation | | | | | | | |

**Table S2.** of Frequency Domain Features Utilised in Previous Studies.

| **Frequency Domain Features** | **LDC** | **Kerr** | **Kuppervelt** | **Montoye** | **Nguyen** | **Ray** | **Zhang** |
|---|---|---|---|---|---|---|---|
| Dominant Frequency | ✓ | ✓ | × | ✓ | ✓ | × | ✓ |
| Power | ✓ | ✓ | × | ✓ | × | × | ✓ |
| Power / Total Power | × | × | × | × | × | × | ✓ |
| Power Dom Freq / Total power (0.3-15 Hz) | × | × | × | × | × | ✓ | × |
| Ratio Dom Freq (0.3-15 Hz) curr/prev windows | × | × | × | × | × | ✓ | × |
| Total Power (0.3-15 Hz) | × | × | × | × | × | ✓ | × |
| Secondary Dom. Freq | × | × | × | × | × | × | ✓ |
| Secondary Power (Dom. Freq.) | × | × | × | × | × | × | ✓ |
| Secondary Dom. Freq (0.3-15 Hz) | × | × | × | × | × | ✓ | × |
| Secondary Power (Dom. Freq) (0.3-15 Hz) | × | × | × | × | × | ✓ | × |
| Dom Freq (0.3-3 Hz) | × | ✓ | × | × | × | × | × |
| Dom Freq (0.3-15 Hz) | × | × | × | × | × | ✓ | × |
| Dom Freq (0.6-2.5 Hz) | × | × | × | × | × | × | ✓ |
| Dom Freq Ratio (current/prev segment) | × | × | × | × | × | × | ✓ |
| Power Dom. Freq (0.3-3 Hz) | × | ✓ | × | × | × | × | × |
| Power Dom. Freq (0.3-15 Hz) | × | × | × | × | × | ✓ | × |
| Power Dom. Freq (0.6-2.5 Hz) | × | × | × | × | × | × | ✓ |
| Power Dom. Freq (1-15 Hz) | × | ✓ | × | × | × | × | × |
| Entropy (freq dom) | × | ✓ | × | × | × | × | × |
| Integral (0.6-2.5 Hz) | × | × | × | ✓ | × | × | × |
| Integral % / Total Integral | × | × | × | ✓ | × | × | × |
| Coefficient Variation | × | ✓ | × | × | × | × | × |
| 1-s lag Autocorrelation | × | ✓ | × | × | × | ✓ | × |
| Theta energy (tilt, angle, time) | × | × | × | × | ✓ | × | × |
| Entropy | × | × | × | × | × | ✓ | × |

**Table S3.** Average cluster purity and event purity.

| **Cluster Purity Across Four Datasets (2 development and 2 lab independent)** | | | | |
|---|---|---|---|---|
| | **Sedentary**<br><br>**Clusters A-E** | **Vigorous**<br><br>**Cluster J** | **Ambulatory (Brisk) Cluster I** | Ambulatory (Slow) Clusters G-H |
| ACP (Average Cluster Purity) | 0.66 | 0.78 | 0.52 | 0.34 |
| AEP (Average Event Purity) Based on Lying+Seated Average | 0.67 | 0.83 | 0.51 | 0.74 |
| ACEP (Average Cluster & Event Purity) | 0.65 | 0.80 | 0.49 | 0.50 |
